# Degenerate seaweed to tilted dendrite transition and their growth dynamics in directional solidification of non-axially oriented crystals: a phase-field study

**DOI:** 10.1038/srep26625

**Published:** 2016-05-23

**Authors:** Hui Xing, Xianglei Dong, Hongjing Wu, Guanhua Hao, Jianyuan Wang, Changle Chen, Kexin Jin

**Affiliations:** 1 The Key Laboratory of Space Applied Physics and Chemistry, Northwestern Polytechnical University, Xi’an, 710129, P.R. China

## Abstract

We report the results of a phase-field study of degenerate seaweed to tilted dendrite transition and their growth dynamics during directional solidification of a binary alloy. Morphological selection maps in the planes of (*G*, *V*_*p*_) and (*ε*_4_, *V*_*p*_) show that lower pulling velocity, weaker anisotropic strength and higher thermal gradient can enhance the formation of the degenerate seaweed. The tip undercooling shows oscillations in seaweed growth, but it keeps at a constant value in dendritic growth. The M-S instability on the tips and the surface tension anisotropy of the solid-liquid interface are responsible for the formation of the degenerate seaweed. It is evidenced that the place where the interfacial instability occurs determines the morphological transition. The transient transition from degenerate seaweed to tilted dendrite shows that dendrites are dynamically preferred over seaweed. For the tilted dendritic arrays with a large tilted angle, primary spacing is investigated by comparing predicted results with the classical scaling power law, and the growth direction is found to be less sensitive to the pulling velocity and the primary spacing. Furthermore, the effect of the initial interface wavelength on the morphological transition is investigated to perform the history dependence of morphological selection.

The selection of crystal growth patterns in solidification has been of long-standing fundamental interests in studies of scientific and engineering significance^1^. The interplay between external macroscopic kinetic (thermal and mass diffusion) and internal microscopic interfacial dynamics (interfacial anisotropy) determines that. Dendrites, resulting from the morphological instability of the solid-liquid interface, are the most frequently observed microstructural patterns from casting to welding. Mechanical properties of product casting strongly depend on the complex patterns created by dendritic network as well as length scales^2^,^3^. Therefore, understanding the formation and evolution of dendritic structures as well as their morphological transition is of particular importance due to major scientific and technological interests. A breakthrough in the understanding of the dendrite growth is that the surface tension anisotropy plays a crucial role in the formation of the dendrite through acting as a singular perturbation to destroy the non-uniqueness of the selected tip^4^,^5^. Besides the surface tension anisotropy, the kinetic anisotropy is also important for the crystal growth in some situations. Ihle^6^ studied the combined effects of the surface tension anisotropy and the kinetic anisotropy on morphological selection as well as the competition between them.

Seaweed, or dense branching morphology, is known as another important class of interface morphologies in solidification^7^,^8^,^9^,^10^. Dendrites are patterns with obvious orientational order, whereas seaweed structures are considered as patterns without orientational order. The defining characteristic of seaweed is the successive and continuous splitting of the tips, which is a common feature of diffusion limited growth of isotropic or weakly anisotropic crystals. Ben-Jacob *et al*.^7^ firstly observed the seaweed morphology during the annealing of amorphous Al-Ge alloys. By an extensive and deep theoretical analysis based on scaling relations, Brener *et al*.^8^ predicted a large class of free growth patterns in two-dimensional diffusion-controlled growth and determined the stability ranges of dendrite and seaweed in the plane of (*ε*, Δ), where *ɛ* is the anisotropy strength and Δ is the imposed undercooling. Subsequently, Singer *et al*.^9^ determined the stability ranges of morphologies of free growth in three-dimensional systems, showing that the dendrite can evolve into doublon or seaweed as the undercooling is increased. Indeed, it is evidenced that noise at the solid-liquid interface is another crucial factor to drive the transition to the seaweed since the noise with sufficient intensity not only results in the emission of the sidebranches but also destroys the stability of the growing tip. By comparing the real-time observation by the X-Ray radiography with the numerical simulations, Chen *et al*.^10^ provided direct evidence of the transition from dendritic or cellular to seaweed structures in solidification of Al-Cu alloys. Their numerical results show that when the noise, whatever the physical or numerical origin, is enough large, the dendrite-to-seaweed transition can occur even if the [010] crystallographic direction of preferred growth for cubic primary-Al solid is aligned with the thermal gradient direction. Although the seaweed structures in their work are in transient and the microstructures are eventually achieved in steady-state dendritic growth, it can be proved that favorable conditions for the seaweed structures can be obtained in practice particular cases.

Directional solidification of alloys has attracts significant attentions over several decades as a well-established paradigm for the investigation of competitive effects and the arising morphologies in non-equilibrium systems with technological importance^1^,^2^,^3^. In this process, the alloys sample is solidified through a fixed temperature gradient *G* with the pulling velocity *V*_*p*_. Combined with the theoretical findings, previous experiments and simulations have shed light on some of the fundamental aspects of the growth of dendritic arrays, such as the selection of tip radius^11^,^12^,^13^, the range of stable primary spacing^14^,^15^,^16^, the mechanism of the wavelength selection^17^,^18^, the selection of growth direction^19^,^20^, and the growth competition between columnar grains^21^,^22^. Seaweed structures were also reported in directional solidification of cubic crystals at a low anisotropy system. Experimental^23^,^24^,^25^,^26^,^27^ and numerical^28^,^29^,^30^ studies have been carried out to prove the stability, characteristics and formation of seaweed structures over last decades. According to the growth patterns observed in experiments, Akarmatsu *et al*.^24^ distinguished two types of seaweed structures, seaweed pattern and degenerate pattern (degenerate seaweed), in directional solidification. The seaweed pattern, which is related to the weak anisotropy, can be considered as a true permanent regime in directional solidification when grains are pretty close to the {111} plane. They found that the nature of the seaweed pattern is the permanency of its morphology over a wide range of pulling velocity. Degenerate pattern, or degenerate seaweed, is another striking type of seaweed in directional solidification., originates from the competition between the heat flow direction established by the applied thermal gradient and the preferred growth direction resulting from the surface tension anisotropy. In cubic crystals, two <100> directions are symmetrical with respect to the direction of thermal gradient. The degenerate seaweed is unsteady and strongly disordered at low pulling velocity. As the pulling velocity is increased, the solidification front undergoes a transition from degenerate seaweed to tilted dendrite. The formation of seaweed patterns is inherently related to the low anisotropy because constraining the crystal growth to specific orientations is difficult in this case. Utter *et al*.^25^ investigated the growth dynamics of low anisotropic crystals in directional solidification by discussing the tip-splitting instability of the seaweed. The quasi-periodic behaviors of tip-splitting in the growth of degenerate seaweed were analyzed by a simple model that assumes the appearance of tip-splitting once the tip becomes sufficiently flat^26^. Of particular importance in their experiments^25^,^26^ is that there exists a morphological transition from degenerate seaweed to tilted dendrite as the thermal gradient is decreased. This is qualitatively confirmed by subsequent numerical simulations^28^. However, the precise mechanism of the transition from degenerate seaweed to tilted dendrite and their growth dynamics are less clearly understood.

In this study, we would like to discuss the scaling laws for the selection of morphological transition, cell spacing of the degenerate seaweed, primary spacing and growth direction of tilted dendritic arrays with a large tilted angle as well as the dynamic mechanism of the degenerate seaweed to tilted dendrite transition. The following questions are specially addressed:

- Previous work^24^,^25^,^26^,^28^,^29^,^30^ has confirmed that a morphological transition from degenerate seaweed to tilted dendrite occurs as the thermal gradient is decreased or the pulling velocity is increased. However, the mechanism of this transition and the dynamic characteristics of degenerated seaweed, especially the relationship between the tip-splitting instability and the sidebranching, are relatively less studied.
- Primary spacing of dendritic arrays in directional solidification follows a modified power law by Gandin *et al*.^31^ for tilted growth. Whether the primary spacing of dendritic arrays with a larger tilted angle can be scaled by the same power law is unclear.
- Utter *et al*.^25^ observed that the growing tip is tilted from the thermal gradient direction beyond 45°, which is very interesting because the largest misorientation angle for cubic crystals is 45°. Can we reproduce the dendritic arrays with a tilted angle beyond 45° in numerical simulations? Whether the growth direction follows the growth direction selection law for the tilted growth of dendritic arrays by Deschamps, Georgelin and Pocheau (DGP)^19^ is still unclear.
- For the growth of dendritic arrays, a similar history leads to a similar primary spacing^15^,^16^. Similarly, for degenerate seaweed, what is the influence of the initial wavelength of the interface on the selection of growth patterns?

Numerical simulation is a possible way to clarify the transition from degenerate seaweed to tilted dendrite and their growth dynamics. The phase-field method has been a powerful tool for simulating the evolution of complex interfacial morphology in recent years^32^. It has become more quantitative with respect to its conformity to the well-known sharp-interface model when the so-called thin-interface limit^33^ is implemented. In this limit, the physical dimensions of the interface can be many times larger than the capillary length, and hence it dramatically increases the corresponding length and time scales. For binary alloys solidification, a breakthrough in the thin-interface limit is that the added anti-trapping term not only recovers local equilibrium at the interface but also eliminates spurious effects appearing when the use of the interface width is larger than the capillary length^34^,^35^. Plenty of investigations on microstructure evolution of solid-liquid interface morphology under various solidification conditions^36^,^37^,^38^,^39^,^40^ have been carried out by using the phase-field model. A first step using the phase-field model to quantitatively investigate the mechanism of the transition from degenerate seaweed to tilted dendrite in directional solidification was taken by Provatas *et al*.^28^. In their work, predicted results show that the morphology of the transition from seaweed to dendrite can be unambiguously characterized by the broading of local interface velocity distribution. Furthermore, they derived a semi-analytical theory of this transition, yielding a morphological diagram in the (*G*, *V*_*p*_) plane for lower pulling velocity. Amoorezaei *et al*.^29^ investigated the morphological transition in Mg alloys with hexagonal close-packed lattice, suggesting that the arising morphologies such as tilted dendrites, fractal and compact seaweed structures from various solidification conditions are more complex than previous thought. Subsequent studies by Tourret and Karma^39^ show that the larger thermal gradient and lower misorientation angle can trigger the transition from seaweed to dendrite. In additions, the influence of interplay of different sources of anisotropies on the orientation selection and the resulting microstructures has been reported in equiaxed dendritic growth^41^ and directional solidification of dendritic arrays^42^. Results show that the anisotropy parameters correspond to the hyperbranched region that produces complex degenerate seaweed structures^42^.

To address the questions presented above, the thin-interface phase-field method^35^ is adopted to simulate the directional solidification of an alloy for investigating the mechanism of transition from degenerate seaweed to tilted dendrite and their growth dynamics. In order to obtain low anisotropy in the direction of the thermal gradient, the angle between the thermal gradient direction and crystalline anisotropy is set at 45° for the cubic crystals. In particular, effects of the thermal gradient, pulling velocity, and crystalline anisotropy on the selection of solidification patterns are investigated. The mechanism of the degenerate seaweed to tilted dendrite transition as well as their growth dynamics are discussed by correlating the tip-splitting instability to the emission of sidebranches. The characteristic of the tilted dendrites with a large tilted angle is also discussed by comparing predicted results with the scaling power law. Furthermore, the history dependence of the morphological transition is discussed by changing the initial wavelength of the solid-liquid interface.

## Phase-field model and computational procedures

The thin-interface phase-field formulation for directional solidification introduced by Echebarria *et al*.^35^ is adopted here to investigate the transition from degenerate seaweed to tilted dendrite during directional solidification of a binary alloy. This model has been successfully used in several alloys solidification under various conditions^28^,^29^,^30^,^37^,^38^,^39^,^40^,^41^,^42^. Detailed derivations and validations of this model can be found in the references provided above. Here we present the equations describing the evolution of the phase-field variable and concentration field as well as a brief introduction of the numerical domain and procedures. Al-3wt% Cu is chosen as the numerical calculation object, and all materials properties are assumed constants. We neglect the diffusion in the solid, which allows simplification to a one-sided model. After neglecting the latent heat production, a so-called “frozen temperature approximation” is adopted for the directional solidification issues
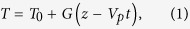
where *T*_0_ is a reference temperature, the temperature gradient *G* is aligned with the *z* direction, and the sample is pulled with a constant pulling velocity *V*_p_. Furthermore, the convection in the liquid is also neglected because it is weak in thin sample experiments. In this model, a scalar field *ϕ*(***r**,t*) is introduced to determine the phase at the fixed point and time. The field *ϕ*(***r**,t*) takes on the values *ϕ* = 1 in the solid phase, *ϕ* = −1 in the liquid phase and varies continuously across the interface. The concentration field *c*(***r**,t*) is characterized by a generalized supersaturation field *U* with respect to (*c*_*l*_^0^,*T*_0_). This generalized supersaturation field *U* is given by *U* = ((2*kc*/*c*_∞_)/[1 + *k*−(1−*k*)*ϕ*]−1)/(1−*k*), where *c*_∞_ is the average solute concentration, *k* is the partition coefficient and *c*_*l*_^0^ = *c*_∞_/*k* is the concentration on the liquid side of the interface. For cubic crystals, a four-fold anisotropy is implemented here, and the anisotropy function in two-dimensional system can be expressed as
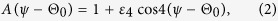
where *ε*_4_ is anisotropic intensity of the surface tension for cubic crystal, *ψ* is the angle between the interface normal and the axis *z*, and Θ_0_ is the angle between the preferred crystalline orientation and the direction of thermal gradient, the misorientation angle. It should be noted that an expression of the anisotropy in three-dimensional system given by Haxhimali *et al*.^41^ is slightly different from the above expression in two-dimensional system. It has been confirmed that their expression of anisotropy admits <100>, <110> and <111> preferred directions in three-dimensional systems. The competition between different preferred directions in three-dimensional system with the resulting growth patterns needs further investigations in future work.

The equations of motion for phase-field and generalized supersaturation field are given by
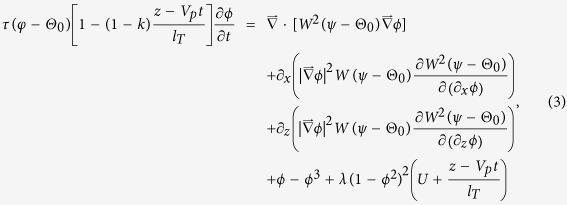

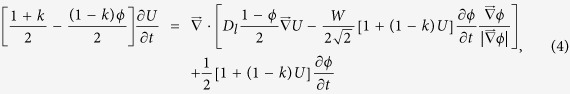
with 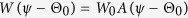,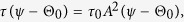 where *λ* is the coupling constant between the two equations, *l*_*T*_ = |*m*|*c*_∞_(1−*k*)/(*kG*) is the thermal length, *d*_0_ = *Γ*/Δ*T*_0_ is the capillary length, Δ*T*_0_ = |*m*|(1−*k*)*c*_*l*_^0^ is the freezing range, *m* is the liquidus slope, *Γ* is the Gibbs-Thomson coefficient, *l*_*D*_ = *D*_*l*_/*V*_*p*_ is the solute diffusion length. *τ*_0_ and *W*_0_, representing a relaxation time and a measure of interface width, respectively, are the time and length scales. In this work, we neglect the kinetic effect, i.e., *β*_*k*_ = 0, at least to first order. *τ*_0_ and *W*_0_ can be linked with physical properties by two relations. The capillary length can be expressed in terms of the phase-field parameters *W*_0_ = *d*_0_*λ*/*a*_1_, and the requirement of vanishing interface kinetics to the lowest order yields *τ*_0_ = *a*_2_*λW*_0_^2^/*D*_*l*_, where *a*_1_ = 0.8839 and *a*_2_ = 0.6267 are numerical constants depending on the choices of the free energy function. The interface width is given by *W*_0_, and hence *λ* is seen as the numerical convergence parameter of this model. We solved Equations (3) and (4) in a two dimensional system using the explicit finite difference method on a fixed grid. A standard nine-point stencil Laplacian operator is used in simulations. Al-3 wt.% Cu alloy is adopted in the present investigations, and the properties used in simulations as follows: *m* = −2.6 K/wt. %, *D*_*l*_ = 3000 μm^2^/s, Γ = 0.24 K∙μm, *k* = 0.14, and *ε*_4_ = 0.01 unless otherwise noted. To increase computational accuracy, the mesh spacing is set as Δ*x* = 0.4. Solidifications were initialized with a thin solid layer. The initial phase-field variable is set as *ϕ* (*z*,0) = tanh(*z*/√2) along the normal to the planar interface while the initial supersaturation field *U* is set to a steady-state diffusion profile along the normal to the interface.

**Figure 1:**
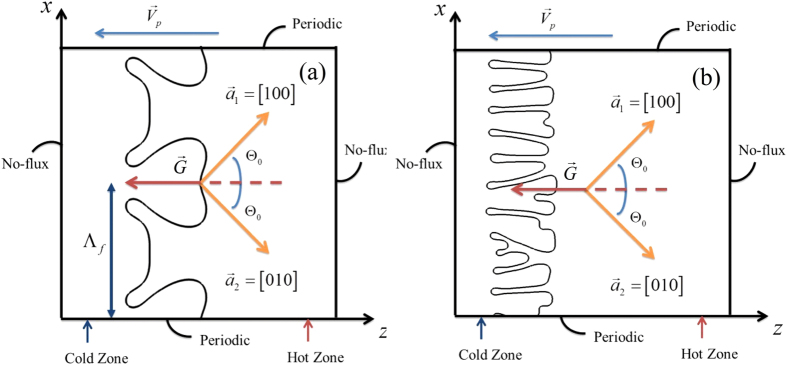
Schematic of the crystal growth during directional solidification of non-axially oriented crystals, (**a**,**b**) show the initial growth patterns from simulations that are initialized using randomly noise with small amplitude on a flat interface and a flat interface except for small perturbations with an interfacial wavelength, respectively, where 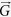 and 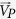 are the thermal gradient and the pulling velocity, respectively, 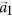 and 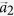 present the two preferred crystalline directions for this grain, the misorientation angle Θ_0_ is fixed at Θ_0_ = 45°, Λ_*f*_is the initial interfacial wavelength.

The precision of the results increases as *W*_0_ decreases. However, decreasing *W*_0_ means a dramatic increase in the computational cost of the simulation. As discussed in Reference^22^, results from simulations do not appreciably depend on the interface width as long as it remains to be one order of magnitude smaller than the characteristic length scale of the problem. In directional solidification, the accuracy of the numerical calculation depends not only on the pulling velocity but also on the anisotropic strength. In this study, in order to determine the largest possible interface width *W*_0_ or the coupling constant *λ*, a systematic convergence study should be carried out before numerical simulations. For the convergence study, the solid-liquid interface is initialized as a planar except for small perturbations with a wavelength Λ_*f*_ (the wave number *Q* = 2π/Λ_*f*_) and the amplitude *A*_*p*_ (Fig. 1(a)). Due to the history dependence of growth patterns, the final morphologies also depend on the choice of the initial interface. In the convergence study the initial wavelength is set as a constant.

**Figure 2:**
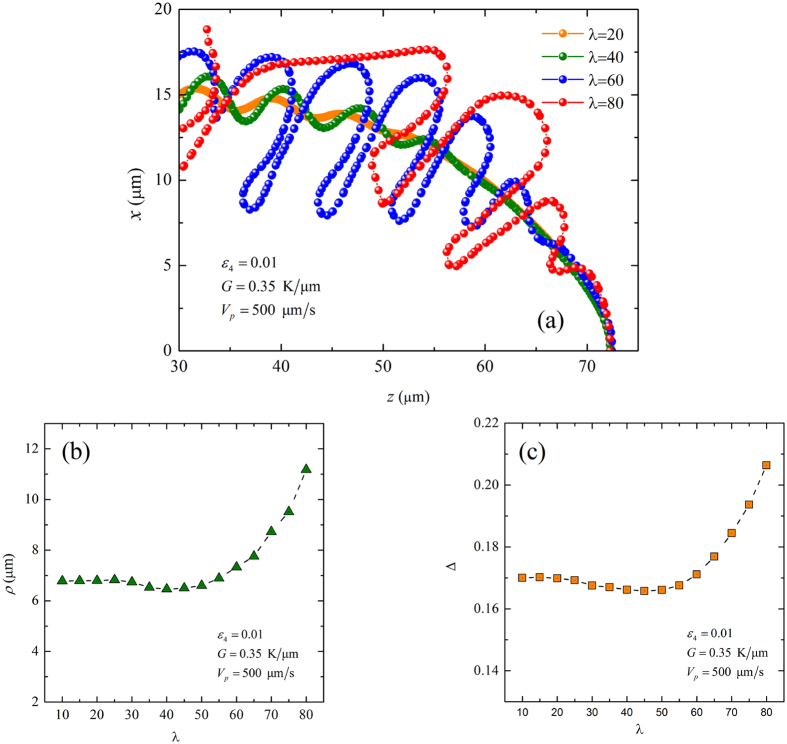
(**a**) Interface contours of steady-state dendrite growth for different values of λ; tip radius (**b**) and dimensionless tip undercooling (**c**) with respect to λ, where *G* = 0.35 K/μm, *V*_*p*_ = 500 μm/s, Λ_*f*_ = 36.2 μm and *ε*_4_ = 0.01.

Although artificial noise is not added in our simulations, the residual grid noise may have an influence on the growth pattern selection. Therefore, before the convergence study, it is necessary to qualitatively show the relationship between the residual grid noise and the coupling constant *λ*. Because it is difficult to observe the strength of the grid noise in the degenerate seaweed growth and the tilted dendritic growth, the growth of a dendrite with Θ_0_ = 0° in directional solidification is first performed. Figure 2(a) presents the interface contours of the steady-state dendrite in directional solidification for four different values of the coupling constant *λ*, where *G* = 0.35 K/μm, *V*_*p*_ = 500 μm/s, Λ_*f*_ = 36.2 μm and *ε*_4_ = 0.01. It can be seen that the amplitude of the sidebranches of the dendrite dramatically increases with the increase of *λ*, which means that the residual grid noise increases with the increase of *λ*. Obviously, the increase of *λ* not only results in the increase of the amplitude of the sidebranches, but also has an influence on the shape of the dendritic tip. For *λ* ≤ 40, the contours are convergent at the tip region. As *λ* increases, the place of the first sidebranching approaches towards the dendritic tip with the change of the tip shape. To better describe it, the tip radius *ρ* and the dimensionless tip undercooling Δ versus *λ* are plotted in Fig. 2(b,c), respectively, where the dimensionless undercooling Δ = (*T*_*l*_−*T*_*t*_)/Δ*T*_0_ = 1−*z*_*t/*_*l*_*T*_ is used to determine the tip position. Δ*T*_0_ = *T*_*l*_−*T*_s_ = |*m*|(1−*k*)*c*_*l*_^0^ is the equilibrium freezing temperature range, *T*_*t*_ is the tip temperature, *T*_*l*_ and *T*_*s*_ are the liquidus and solidus temperatures for *c*_∞_, respectively, and *z*_*t*_ is the position of the leading tip, *z*_*t*_ = 0 corresponds to the position of the steady-state planar interface. Both the tip radius and the tip undercooling converge to constant values with the decrease of *λ*. All simulated results show good convergence for *λ* ≤ 40. This study shows that although the residual grid noise may have an influence on the growth pattern selection, decreasing *λ* can reduce the residual grid noise and restrict its influence on the growth patterns, at least near the tip. This is important for the study of the transition from degenerate and dendrite as the simulated results are reasonable as far as the interface width is sufficiently small.

**Figure 3:**
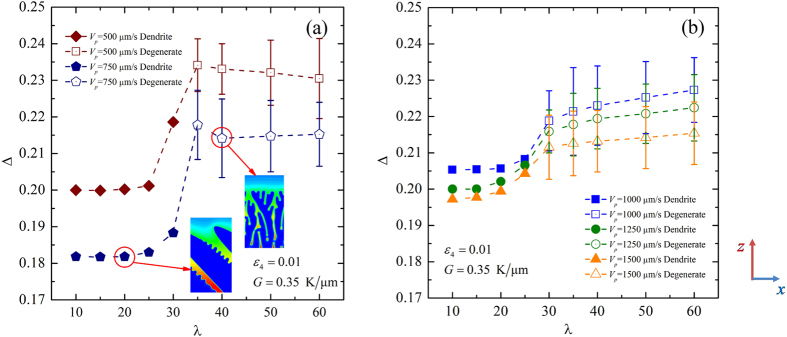
Dimensionless tip undercooling with respect to λ for *V*_*p*_ = 500 μm/s and 750 μm/s when Λ_*f*_ = 36.2 μm (**a**) and *V*_*p*_ = 1000 μm/s, 1250 μm/s and 1500 μm/s when Λ_*f*_ = 18.1 μm (**b**).

**Figure 4:**
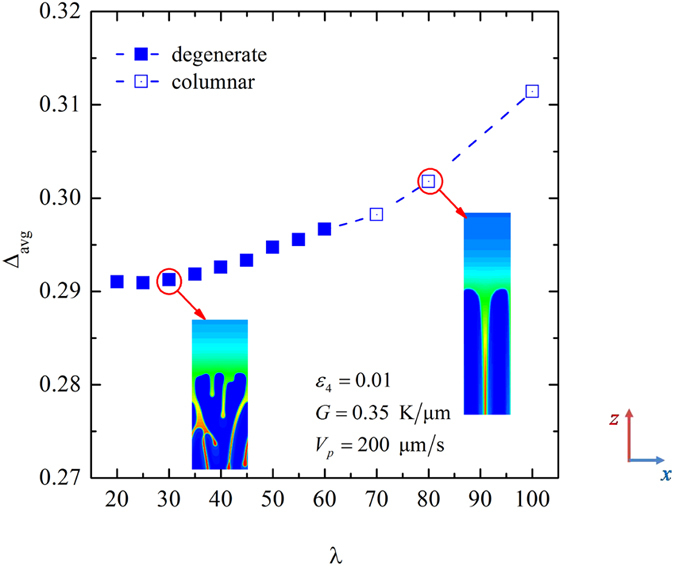
Averaged dimensionless tip undercooling with respect to λ for *V*_*p*_ = 200 μm/s when Λ_*f*_ = 36.2 μm.

We first perform the convergence study for different pulling velocity. In this convergence study, the thermal gradient is fixed at *G* = 0.35 K/μm and the anisotropic strength is set as *ε*_4_ = 0.01. Because the stable range of the primary spacing is varied with the variation of the pulling velocity for a fixed the thermal gradient, the values of the initial wavelength Λ_*f*_ are different in different cases of pulling velocity. Here, we focus on the convergence of the dimensionless tip undercooling with respect to *λ*. The dimensionless tip undercooling versus *λ* as well as typical growth patterns for *V*_*p*_ = 500 μm/s and 750 μm/s when Λ_*f*_ = 36.2 μm are plotted in Fig. 3(a). The solid symbols correspond to dendritic patterns while the empty symbols correspond to the degenerate seaweed. Because the dimensionless tip undercooling oscillates with time in the seaweed growth, the error bars can be used to represent the range of its variation. It can be seen that with the decrease of *λ*, there is a transition from degenerate seaweed to tilted dendrite, meanwhile the value of the dimensionless tip undercooling eventually converges to a constant for *λ* ≤ 20. Here, the morphological transition is due to the decrease of the residual grid noise with the decrease of *λ*. It implies the importance of noise in the morphological transition as the morphologies are sensitive to any kind of noise when Θ_0_ = 45°. The above convergence study reveals that the growth patterns are independent of the interface width for *V*_*p*_ = 500 μm/s and 750 μm/s when *λ* ≤ 20. However, for a larger pulling velocity, the chosen interface width should be smaller in order to satisfy the constraint that growth patterns are independent of *λ*. Figure 3(b) shows the dimensionless tip undercooling versus *λ* for *V*_*p*_ = 1000 μm/s, 1250 μm/s and 1500 μm/s when Λ_*f*_ = 18.1 μm. Similarly, the transition from degenerate seaweed to tilted dendrite occurs and the value of the dimensionless tip undercooling converges to a constant with the decrease of *λ*. According to simulated results, we choose the coupling constant *λ* = 20, 15 and 10 for *V*_*p*_ = 1000 μm/s, 1250 μm/s and 1500 μm/s, respectively. Until now, we have presented the convergence study when *V*_*p*_ ≥ 500 μm/s, and the dimensionless tip undercooling works for these tests. However, for a smaller pulling velocity, when the degenerate seaweed is independent of the interface width, the dimensionless tip undercooling does not work for the convergence study anymore since the difference among them is very small, as shown in Fig. 3(a,b). Therefore, for *V*_*p*_ = 200 μm/s, we average the dimensionless tip undercooling over one million time steps for different values of *λ*. Figure 4 shows the averaged dimensionless tip undercooling with respect to λ for *V*_*p*_ = 200 μm/s when Λ_*f*_ = 36.2 μm. For larger values of λ, the growth patterns become steady columnar. This type of growth pattern depends strongly on the chosen coupling constant. With the decrease of λ, growth patterns become the degenerate seaweed, and the averaged dimensionless undercooling nearly converges to a constant for *λ* ≤ 30. Because the convergent zone for *V*_*p*_ = 100 μm/s is larger than that for *V*_*p*_ = 200 μm/s, *λ* = 30 can be used in the cases of both *V*_*p*_ = 100 μm/s and 200 μm/s to obtain the growth patterns that are independent of *λ*. The coupling constant *λ* and the interface width *W*_0_ used in the phase-field simulations of the morphological transition in the space of (*G*, *V*_*p*_) are shown in Table 1.

**Figure 5:**
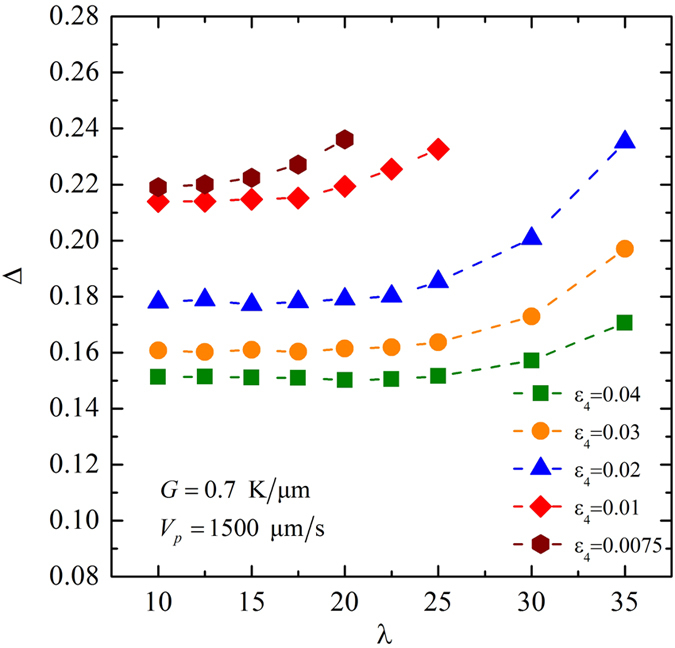
Dimensionless tip undercooling with respect to the coupling constant λ for different anisotropic strength, where *G* = 0.70 K/μm, *V*_*p*_ = 1500 μm/s, and Λ_*f*_ = 18.1 μm.

Let us turn to the convergence study for different anisotropic strength. For the investigation of the morphological transition in the space of (*ε*_4_, *V*_*p*_), the thermal gradient is fixed at *G* = 0.70 K/μm. As simulated results shown in Figs 3 and 4, the convergent zone for a lower pulling velocity is larger than that for a larger pulling velocity. If the chosen interface width can be used in the cases of the largest pulling velocity, it certainly can be used in others cases of lower pulling velocity. Hence, a convergence study for *ε*_4_ = 0.0075 ~ 0.04 is carried out when *V*_*p*_ = 1500 μm/s, which is the largest pulling velocity used in this study. Figure 5 shows the dimensionless tip undercooling with respect to the coupling constant *λ* for different anisotropic strength in the dendritic regime. In all cases of anisotropic strength, Δ increases with the increase of *λ*. But different values of anisotropic strength have different convergent behaviors. For stronger anisotropy (*ε*_4_ = 0.02 ~ 0.04), the dimensionless tip undercooling shows good convergence for *λ* ≤ 20. For the lower anisotropy (*ε*_4_ = 0.01 and 0.0075), *λ* = 10 can be used in simulations to ensure that the growth patterns are independent of the interface width. The coupling constant *λ* and the interface width *W*_0_ used in the investigations of the morphological transition in the space of (*ε*_4_, *V*_*p*_) are shown in Table 2.

Details of our studies are described as follows. Firstly, for the investigation of the morphological transition originating from the initial planar instability, simulations are initialized using random noise with small amplitude on a flat interface, and degenerate seaweed structures or tilted dendritic arrays are allowed to grow from randomly perturbations on the planar interface. Generally, when the pulling velocity *V*_*p*_ exceeds a threshold value *V*_*c*_, the solid-liquid interface undergoes the Mullins-Sekerka instability^43^ and the perturbations will become seaweed structures or dendrites. Figure 1(b) schematically shows that the initial growth patterns from simulations that are initialized using randomly noise with small amplitude on a flat interface, where ***a***_**1**_ and ***a***_**2**_ present the two preferred crystalline directions for a cubic crystal, ***G*** and ***V***_***p***_ are thermal gradient and pulling velocity, respectively, the misorientation angle Θ_0_ is fixed at 45°. The bottom and top boundaries are treated periodically to reduce the domain size and no-flux boundary conditions are applied to the left and right boundaries for both *ϕ* and *U*. In all simulations, the moving-domain technique is implemented following Badillo and Beckermann^37^ to reduce the need for a large computational domain. In our simulations, the lowest pulling velocity *V*_*p*_ is at 100 μm/s, and hence the largest solute diffusion length is *l*_*D*_ = 30 μm. Once the computation domain ahead of the dendrite tip is too short compared with the solute diffusion length, the morphological selection is affected. To avoid that, the size of computational domain ahead of the tip is set as 1000 grid points that are far more than the solute diffusion length. Moreover, the lateral width of the computational domain is set as 3000 grid points that are larger than both the primary spacing of dendritic arrays and the cell spacing of degenerate seaweed. With the moving-domain technique and periodic boundary condition, the number of grid points used in simulations is 3000 × 3000 for the first class of simulations. Secondly, for the investigation of the history dependence of the morphological transition, the grain is allowed to grow from periodic perturbations on the planar solidification front. The solid-liquid interface is initialized as planar except for small perturbations with a wavelength, Λ_*f*_, as schematically shown in Fig. 1(a). In this case, different values of the initial wavelength are simulated by keeping the interface width fixed and varying the size of the simulation domain, Λ_*f*_ = *h*_*x*_/2, where *h*_*x*_ is the length of the *x*-axis. Therefore, with the moving-domain technique, the number of grid points in the *x*-axis in simulations varies with the choice of the initial wavelength and the number of grid points in the *z*-axis is set to 3000.

## Results and Discussions

While previous experiments^24^,^25^,^26^,^27^ were carried out in a thin sample, the thickness of the sample is still larger than the cell spacing of degenerate seaweed and the primary spacing of dendritic arrays, and hence the growth of degenerate seaweed and tilted dendrite is fully three-dimensional and any difference in the tip shape may affect the simulated results. The difference between two-dimensional and three-dimensional numerical results has been discussed in Reference^30^,^40^. Naturally, three-dimensional directional solidification simulations can exhibit the richness of the phenomena, as shown in Reference^41^. However, the full three-dimensional phase-field study of the seaweed and dendritic growth in directional solidification still remains a computational challenge. Indeed, previous two-dimensional numerical studies by Chen *et al*.^10^, Li *et al*.^22^, Akamatsu *et al*.^24^, and Provatas *et al*.^28^,^29^,^30^ can significantly improve our extant understanding of directional solidification of dendritic and seaweed growth. Similar to previous studies, we expect that our two dimensional phase-field simulations can provide qualitatively, or even semi-quantitative, insight into this problem.

Previous investigations^15^,^16^,^40^ offer insight into the effect of the competition between the thermal gradient direction and the crystalline orientation on the resulting morphologies and dendritic growth direction selection for lower misorientation angles. The DGP (Deschamps, Georgelin and Pocheau) law^15^, describing a quantitative relationship between the growth direction and the primary Péclet number, has been established by experiments of succinonitrile (SCN) based alloys, and validated by the numerical simulations^40^. Moreover, it is evidenced that the primary spacing of dendritic arrays can also be affected by this competition^31^. For a larger misorientation angle, generally larger than 40°, the stability of the leading tip is broken, and hence the degenerate seaweed may occur. Naturally, the dendrite to degenerate seaweed transition can be influenced by the misorientation angle, as experimental investigations in Reference^24^ and numerical simulations in Reference^39^.

Very recently, Amoorezaei *et al*.^29^ performed a morphological selection map of directional solidification of Mg-Al alloys in the space of (*G*, *V*_*p*_) from two-dimensional phase-field simulations, where the misorientation angle is fixed at Θ_0_ = 30°, the largest misorientation angle in hexagonal crystals. They identified several main regimes in the morphological selection map: anisotropy directed dendrites (tilted dendrite), degenerate seaweed, and fractal and compact seaweed. Since the anisotropic strength is rather small in Mg-Al alloys, the growing dendrite cannot follow an individual direction and two preferred growth directions can be observed, as shown in Fig. 1 of Reference^29^. They mainly focused on an overall description of the growth pattern selections in the space of (*G*, *V*_*p*_) in this alloy. Details of the transition from degenerate seaweed to tilted dendrite are limited. Moreover, the mechanism of the degenerate seaweed to tilted dendrite transition is not clearly clarified, and the dynamical characteristics of the two growth patterns are not included. Motivated by their finding, we concentrate on this morphological transition, i.e., the transition from the seaweed to anisotropy directed dendrite presented in Fig. 1 of Reference^29^, and simulate the directional solidification of a simple cubic crystals with a fixed misorientation angle Θ_0_ = 45° in two-dimensional system for performing detailed morphological transition in the spaces of (*G*, *V*_*p*_) and (*ε*_4_, *V*_*p*_) and discussing the mechanism of this transition as well as their growth dynamics.

Firstly, a resulting morphological selection map in the plane of (*G*, *V*_*p*_) will be plotted, where the range of pulling velocity is *V*_*p*_ = 100 ~ 1500 μm/s, and the range of the thermal gradient is *G* = 0.14 ~ 1.4 K/μm. The effect of the anisotropy strength on the morphological transition will be also discussed. Secondly, the mechanism of the transition from degenerate seaweed to tilted dendrite will be investigated according to present simulated results. Thirdly, the selections of primary spacing and growth direction of the tilted dendrtic arrays with a large tilted angle will be discussed. At last, for the investigation of history dependence of the morphological transition, the morphological transition with initial interfacial wavelength will be performed for *V*_*p*_ = 500 μm/s and 750 μm/s at a fixed thermal gradient *G* = 0.7 K/μm.

**Figure 6:**
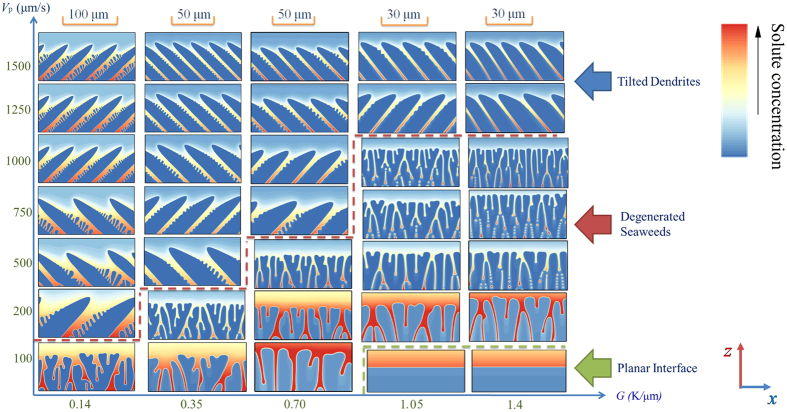
Morphological selection map of Al-3.wt % Cu for Θ_0_ = 45° in the (*G*, *V*_*p*_) plane. The color bar represents the solute concentration and the dashed lines estimate the boundary between the different regimes, the red one is the estimated boundary between the tilted dendrite and the degenerate seaweed while the green one is the boundary between the degenerate seaweed and the planar interface. The scale for each columnar of morphologies is shown on the top of the columnar.

Figure 6 shows the resulting morphological selection map in the space of (*G*, *V*_*p*_) obtained through present phase-field simulations when the anisotropy strength is fixed at *ε*_4_ = 0.01. Three main regimes of the morphologies can be found in this figure: planar, degenerate seaweed, and tilted dendritic arrays. This kind of seaweed structures is called “degenerate” because of a small amount of degeneracy in growth dynamics. Because the transitions are gradual, the sharp limit cannot be identified between these regimes. The main regimes of the resulting morphologies are separated by two color dashed lines: the red one is the estimated boundary between tilted dendrite and degenerate seaweed while the green one is the estimated boundary between degenerate seaweed and planar interface. At velocities just above the Mullins-Sekerka instability^43^ (green dashed line), the planar-to-degenerate seaweed transition occurs and the morphologies become cellular-like, as shown in the (1.05 K/μm, 200 μm/s) and (1.4 K/μm, 200 μm/s) of Fig. 6. The morphologies in the degenerate seaweed regime are characterized by successive and continuous tip-splitting which leads to the alternation of the leading tip. Although the leading tip in the growing degenerate seaweed is altered with time, their growth directions always follow the direction of the thermal gradient. As discussed in Reference^25^,^26^, although the instability occurs on the tips instead of the planar interface, the M-S instability should still be responsible for that because the instability wavelength is linearly related to the M-S instability wavelength. Higher thermal gradient favors the degenerate seaweed, whereas lowering the thermal gradient results in the growth of the seaweed locked into the two specific directions. As the pulling velocity is increased close to the red dashed line, the growth direction has the trend to deviate towards the preferred growth direction until the degenerate-to-dendrite transition occurs, as shown in the (0.14 K/μm, 100 μm/s), (0.35 K/μm, 200 μm/s), (0.70 K/μm, 500 μm/s), (1.05 K/μm, 1000 μm/s), and (1.4 K/μm, 1000 μm/s) of Fig. 6. It can be seen that in these cases, the branches of the degenerate seaweed resemble to the sidebranches of dendrite but the main difference between them is the stability of the leading tip. For the dendrite, the growing tip is stable and the sidebranching events usually occur at a distance behind the tip of about 5–10 *ρ*, where *ρ* is the radius of the dendritic tip. For this kind of degenerate seaweed, the instability of the interface occurs very close to the leading tip, which leads to the tip-splitting instability during the growth of the degenerate seaweed. At velocities above the red dashed line, the stability of the tip cannot be destroyed and morphologies become dendrite-like with a larger tilted angle, and their growth directions are close to the preferred growth direction. The morphological transition from degenerate seaweed to tilted dendrite is qualitatively consistent with the experimental results by Akamatsu *et al*.^24^ and Utter *et al*.^25^ and numerical simulations by Amoorezaei *et al*.^29^. In this tilted dendrite regime, the surface tension anisotropy is dominant, and their growth directions are less sensitive to the pulling velocity and the thermal gradient although the primary spacing varies with the pulling velocity when the thermal gradient is fixed. Hence, the DGP law for the growth direction selection may not be valid here, which will be discussed latter.

Here, we assume that the surface energy anisotropy is the only factor to interplay with the thermal gradient to govern the growth pattern selections. The reason is that the curvature contribution to the undercooling is nearly two orders of magnitude larger than that of the kinetic attachment in solidification of alloys. This is different from the solidification of the transparent model alloys, such as the SCN^25^,^26^,^27^ and CBr_4_-C_2_Cl_6_ 24 alloys, since the kinetic coefficients in the organic systems are about three orders of magnitude than that in metallic systems. In this phase-field model, the kinetic effects are neglected, or at least to first order. Indeed, as the pulling velocity is increased, both the kinetic effect and the curvature contribution to the tip undercooling increases because the increase of the pulling velocity can reduce the radius of the dendritc tip. This alters the growth of the leading tips toward the two <100> preferred growth directions, resulting in the morphological transition. This also indicates that although the kinetic effects are neglected in our simulations, the degenerate seaweed to tilted dendrite transition can also be achieved because of the surface tension anisotropy.

**Figure 7:**
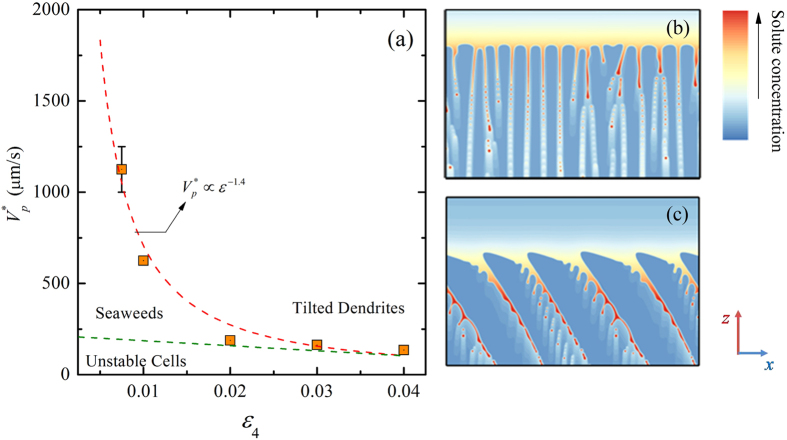
(**a**) Critical velocity for the transition to dendrite versus the anisotropy strength at a fixed thermal gradient (*G* = 0.7 K/μm) and the morphological boundaries in the plane of (*ε*_4_, *V*_*p*_), where the red dashed line represents the boundary between the degenerate seaweed and the tilted dendrite, and the green one represents the boundary between the unstable cells and other growth patterns; (**b**) typical unstable cells at (0.02, 175 μm/s); (**c**) typical tilted dendritic arrays at (0.02, 200 μm/s).

Because the anisotropy plays an important role in the formation of dendritic structures, the degenerate-to-dendrite transition is sensitively dependent on the anisotropy strength. For a cubic system with the same parameters except the anisotropy strength *ε*_4_, we found that the transition from degenerate or unstable cell to dendrite is highly dependent on the anisotropy strength. Figure 7(a) shows that the critical pulling velocity for this morphological transition versus the anisotropy strength at a fixed thermal gradient in the space of (*ε*_4_, *V*_*p*_), where the red dashed line represents the estimated boundary between the degenerate seaweed and the tilted dendrite, and we conjecture the boundary between the unstable cells and other growth patterns (the green dashed line) according to limited simulations for lower pulling velocity. To avoid repetition, morphologies are not plotted in this figure, and typical unstable cellular arrays and dendritic arrays are shown in Fig. 7(b,c), respectively. One can see clearly the critical pulling velocity for the transition to tilted dendrite significantly decreases as *ε*_4_ is increased. For *ε*_4_ = 0.075, the transition occurs at 1125 μm/s that is about 10 times than that for *ε*_4_ = 0.04. It means that the lower the surface tension anisotropy is, the more difficult the transition to dendrite is. Hence, the formation of the degenerate seaweed and the morphological transition to tilted dendrite are closely related to the weak but non-negligible surface tension anisotropy. The critical pulling velocity follows a power-law relationship with respect to the anisotropy strength as *V*_p_^*^∝*ε*_4_^*α*^ with *α* = −1.4. As shown in Fig. 7(b), the morphologies become cellular-like with unstable tips instead of seaweed structures when the pulling velocity is just below the critical pulling velocity (0.02, 175 μm/s). In the cellular arrays, the unstable cells with tip-splitting are rather rare, about less than 10% of cells are unstable. As the pulling velocity exceeds the critical value, the transition from unstable cells (Fig. 7(b)) to tilted dendrite with developed sidebranches (Fig. 7(c)) occurs instead of the transition from unstable cellular arrays to degenerate seaweed to tilted dendrites for *ε*_4_ = 0.01. This indicates that increasing the anisotropy strength can reduce the existence domain of the seaweed structures in the space of (*ε*_4_, *V*_*p*_).

Note that the residual grid noise cannot be completely removed and the residual grid noise can affect the growth patterns as well as the morphological transition although the arbitrary noise is not added in our simulations (except the initial fluctuations at the solid-liquid interface). The residual grid noise results in the formation of the sidebranches of the dendrite when thermal gradient is lower, as shown in Fig. 6. As discussed above, the residual grid noise increases with the increase of the interface width, and even larger residual grid noise can change the place of the first sidebranching and the shape of the tip in dendritic growth. For degenerate seaweed, larger grid noise may have the influence on the events of the tip-instability. In our simulations, relatively smaller interface thickness is chosen in order to reduce the influence of grid noise and obtain the growth patterns that are independent of interface width. The larger the residual grid noise is, the more unstable the solidification front becomes. Therefore, either the increase of the interface thickness or the strength of the added arbitrary noise can lead to that the critical pulling velocity shifts toward higher pulling velocity in Figs 6 and 7(a).

**Figure 8:**
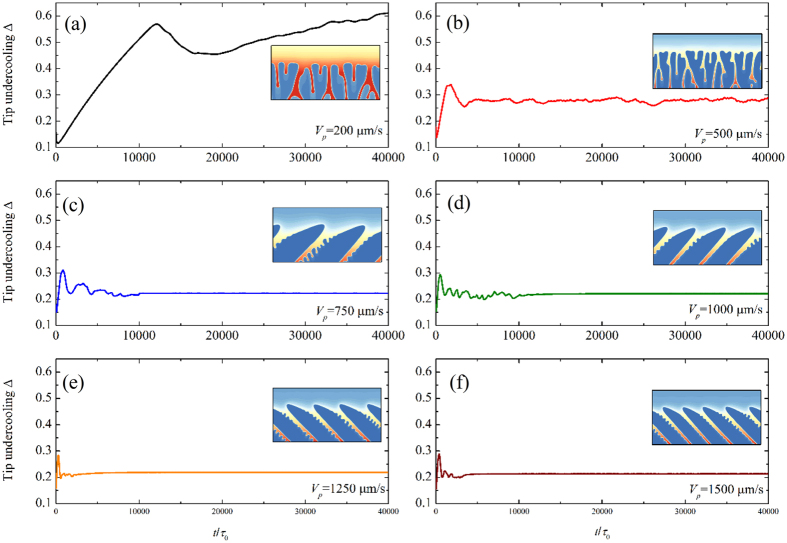
Evolution of the tip undercooling with time with the pulling velocity ranging from 200 μm/s to 1500 μm/s for *G* = 0.7 K/μm.

Compared with the dendrite, degenerate seaweed produced by the competition between the anisotropy and the thermal gradient are more complicated and its growth dynamics is quite different from that of cellular or dendritic growth. The reason is that the events of tip-splitting result in an oscillatory growth dynamics. It is necessary to precisely describe the oscillatory seaweed growth dynamics and the degenerate seaweed to dendrite transition in directional solidification. Numerical simulations by Provatas *et al*.^28^ show that the morphologies of crystal structures can be characterized by the local interface velocity distribution: the broadening of the distribution corresponds to the tilted dendrites while the narrow distribution corresponds to the seaweed. Yet, local interface velocity distribution cannot provide the descriptions of the evolution of the solid-liquid interface from one morphological state to the other. Particularly, it lacks of the description of the oscillatory growth dynamics in seaweed growth. Following the convergence study above, the dimensionless leading tip undercooling Δ can be used to characterize the oscillatory growth dynamics of the degenerate seaweed and distinguish the seaweed mode from the tilted dendritic growth mode. The dimensionless leading tip undercooling as a function of time for six different pulling velocities at a fixed thermal gradient *G* = 0.7 K/μm is displayed in Fig. 8, where the pulling velocity *V*_*p*_ = 200, 500, 750, 1000, 1250, and 1500 μm/s. In order to clearly exhibit the differences, the scales are set to be the same. Except for the case of *V*_*p*_ = 200 μm/s, simulations are continued after the steady-state or the quasi-steady-state is reached. As shown in Fig. 6, the degenerate-to-dendrite transition occurs with the increase of pulling velocity. One can see clearly that the leading tip undercooling eventually shows oscillations in degenerate seaweed growth (Fig. 8(a,b)) while it keeps at a constant value in dendritic growth (Fig. 8(c–f)) after the initial transient stage. The oscillation of the leading tip undercooling implies the competition among neighboring growing cells of the degenerate seaweed and the alternation of the leading tip. Such oscillations still last for a long time (approximately *t*/*τ*_0_ = 10000) until the morphology transfers to tilted dendrite for the cases of *V*_*p*_ = 750 and 1000 μm/s.

**Figure 9:**
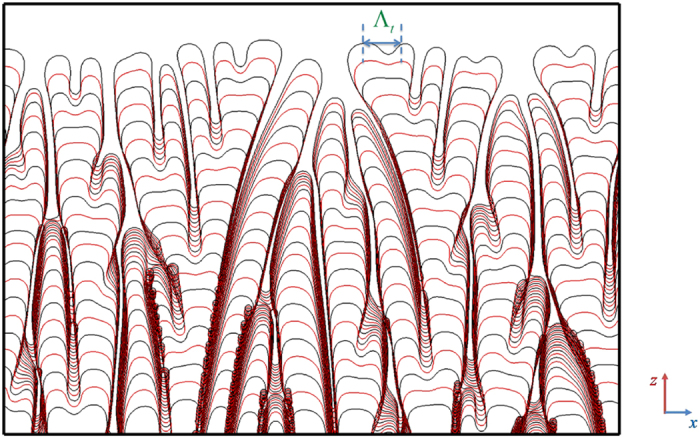
Evolution of the interface morphology with time for *V*_p_ = 500 μm/s and *G* = 0.7 K/μm, where the intersection of the contours *ϕ* = 0 is plotted as the solid-liquid interface with the time interval of Δ*t*/*τ*_0_ = 1000, and Λ_*t*_ shows the spacing between the two advancing cells in the degenerate seaweed structures.

The simulated case with *V*_*p*_ = 500 μm/s at *G* = 0.7 K/μm is a representative example for characterization of degenerate seaweed growth dynamics. The growth mechanism for the degenerate seaweed can be understood by the evolution of the solid-liquid interface with time in Fig. 9, in which the intersection of the contours *ϕ* = 0 is plotted as the solid-liquid interface with the time interval of Δ*t*/*τ*_0_ = 1000. The figure shows that each tip undergoes the same process during the seaweed growth. As the tip becomes unstable, the tip laterally broadens and locally flattens. Then, the flattened tip breaks up and splits into two new tips that tend to grow outwards in two directions near its center. The two asymmetrical main branches produced by two tips with comparable sizes interact with each other until either one falls behind or new tip-splitting occurs. Each tip follows this repetitive process which gives rise to the formation of the degenerate seaweed. Although the anisotropy continuously tries to make the growth of degenerate seaweed deviate from the direction of thermal gradient, the thermal gradient eventually wins this competition and the growth direction still follows the thermal gradient. The main characteristic of the degenerate seaweed in directional solidification is the continuous tip-splitting instability, which results in the growth lacking the apparent preferred orientation of the crystal. The spacing of the two advancing seaweed tips, Λ_*t*_ (see Fig. 9), is of importance to characterize the growth dynamics of the degenerate seaweed. Assume that the tip-splitting instability dynamics is related to the M-S instability, there is an approximately linear relationship between the spacing of the two advancing seaweed tips, Λ_*t*_, and the initial instability wavelength of the planar interface, Λ_*f*_, under the same solidification condition, which yields
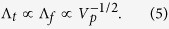


**Figure 10:**
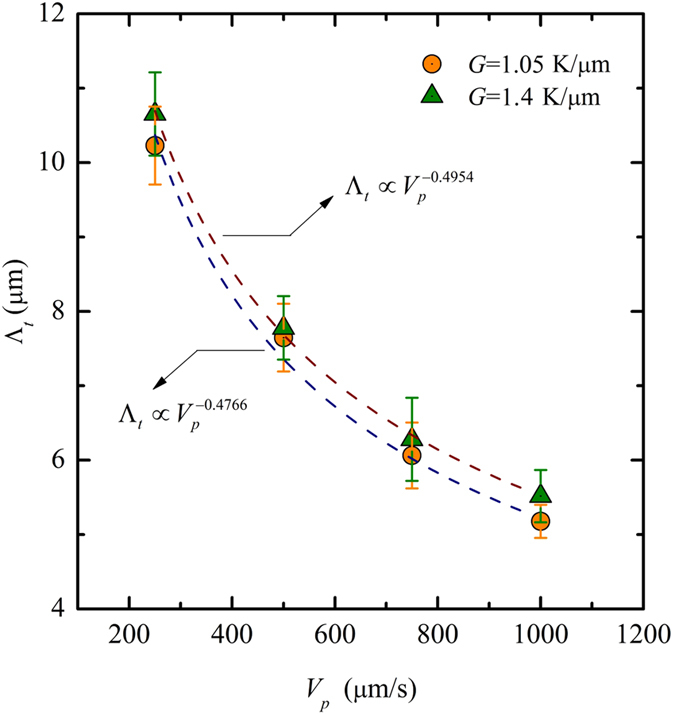
Spacing between the two advancing seaweed tips, Λ_*t*_, as a function of the pulling velocity follows the power law Λ_*t*_∝*V*_*p*_^−*α*^for *G* = 1.05 K/μm and *G* = 1.4 K/μm, where the error bars represent the range of the spacing measured in the same seaweed structures.

Figure 10 shows that the spacing of the two advancing seaweed tips as a function of the pulling velocity follows the power law Λ_*t*_∝*V*_*p*_^−0.4954^ and Λ_*t*_∝*V*_*p*_^−0.4766^ for *G* = 1.05 K/μm and *G* = 1.4 K/μm, respectively. The spacing Λ_*t*_is measured when the distance between the two new tips reaches maximum. We measure all the spacing of the events of tip-splitting over a period of time to determine the range of the spacing Λ_*t*_. The error bars are used to represent the range of the spacing measured in the same structure. The values of exponent −0.4954 and −0.4766 are consistent with the measured instability wavelength of the seaweed tip Λ_*t*_∝*V*_*p*_^−0.5^ by Utter *et al*.^25^ in directional solidification of SCN-PEO alloys, which agrees well with the expected value based on the linear stability analysis of the planar interface. Hence, the tip-splitting instability dynamics of degenerate seaweed is related to the M-S instability dynamics of the planar interface, which means that the instability of the tip-splitting can be considered as the M-S instability on the tip. The weak anisotropy is the reason of the broader of the existing tip, and the M-S instability on the tip results in the occurrence of the tip-splitting instability when the tips flatten. This is the same mechanism found in experiments by Akamatsu *et al*.^24^ and Utter *et al*.^25^,^26^. This mechanism of the tip-splitting instability in the growth of degenerate seaweed is different from that in the finger growth in narrow channels^44^ and the cellular growth when the preferred growth direction is aligned with the thermal gradient direction^45^. While the anisotropy is responsible for the stability of the tip in the latter, it leads to the broader and instability of the tip in the former.

**Figure 11:**
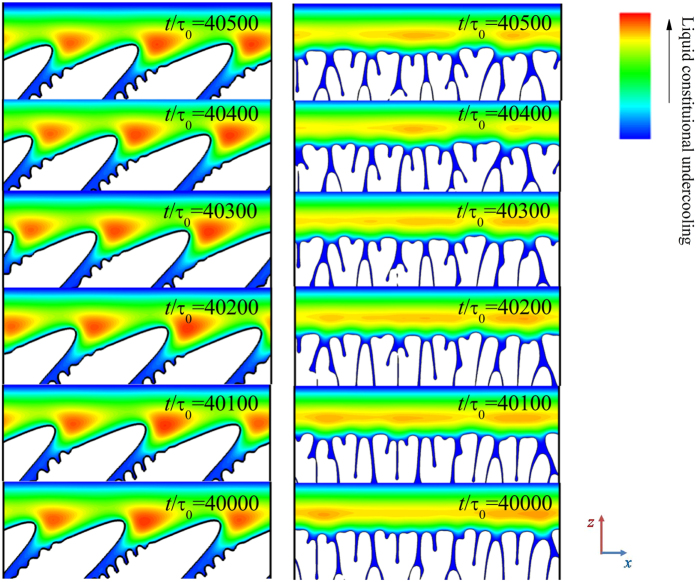
Two representative examples of predicted results of the evolution of the solid-liquid interface morphologies with the distributions of the liquid constitutional undercooling after the quasi-steady-state for seaweed growth for *V*_*p*_ = 500 μm/s (**a**) and the steady-state for dendritic growth for *V*_*p*_ = 750 μm/s (**b**).

It can be seen clearly that the solid grows almost as a planar front during degenerate seaweed growth, which indicates that the dimensionless tip undercooling difference among the tips is rather small. The competition among the growing tips leads to the easy alternation of the leading tip. In order to discuss this competition as well as comparing with the tilted dendritic arrays, the solute concentration in the liquid phase is converted into the constitutional undercooling. The liquid constitutional undercooling, defined as the difference between the liquidus temperature and the actual temperature, i.e., Δ*T* = *T*_*m*_ + *mc*_*l*_−*T*, where *T*_*m*_ is the melting point of pure aluminum, and the actual temperature *T* is given by the imposed temperature, Δ*T* can be seen as the thermodynamic driving force for solidification. Figure 11(a,b) show two representative examples of the evolution of the solid-liquid interface morphologies with the distribution of the liquid constitutional undercooling after *t*/*τ*_0_ = 40000, where the time interval among these figures is Δ*t*/*τ*_0_ = 100. It should be noted that the degenerate seaweed and the dendrite are blank when *ϕ* > 0 because the constitutional undercooling has no physical meaning in solid phase. The undercooled region ahead of the solidification front is consistent with the diffusion boundary layer. Far ahead of the solid-liquid interface, the variation of the constitutional undercooling is linear according to the imposed temperature field because the concentration is at a constant *C*_*0*_. Relatively small constitutional undercooling exists in the liquid intercellular region because the ejected solute is diffused. Relatively larger constitutional undercooling is presented in front of the solidification front, especially the regions between cells or dendrites. During directional solidification, the liquid constitutional undercooling gradient dominates the interface propagation direction, i.e., the largest constitutional undercooling gradient means the fastest growth in this direction. As shown in Fig. 11(a), inhomogeneous distribution of the constitutional undercooling resulting from the complex solidification front leads to the different tips of the degenerate seaweed with different growth speeds. The local maximum constitutional undercooling (red region in these figures) moves in the boundary layer during the degenerate seaweed growth, which results in the shift of the largest constitutional undercooling gradient direction, and hence the growth speeds of the tips are varied with time. As the leading tip changes, the growth speed varies and the dimensionless leading tip undercooling shows oscillations during the degenerate seaweed growth, as shown in Fig. 8(b). In contrast, Fig. 11(b) shows that the local maximum constitutional undercooling always exists ahead the dendritic tip. It is almost stable during the dendritic growth with the growth direction of the tilted dendrite toward the largest constitutional undercooling gradient direction. Hence, the leading tip undercooling (growth speed) of the tilted dendrite is at a constant, as shown in Fig. 8(c).

**Figure 12:**
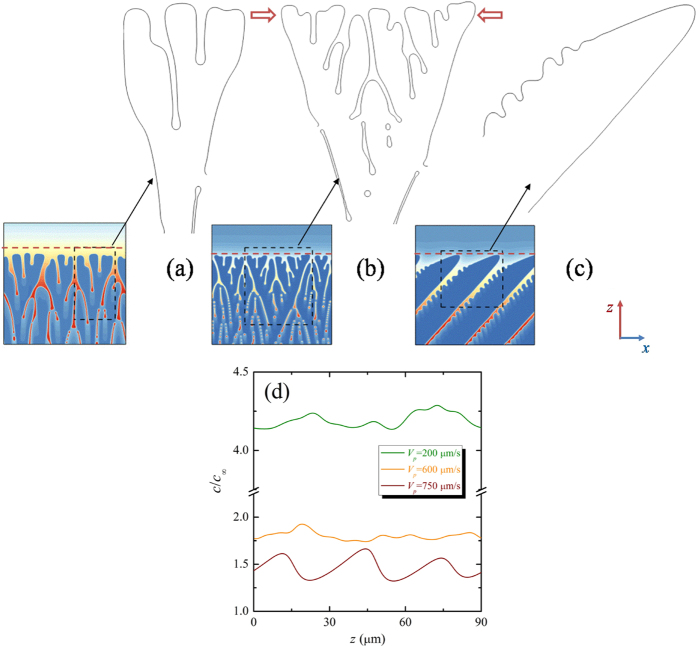
Typical quasi-steady-state interfacial morphologies for *V*_*p*_ = 200 μm/s (**a**), 600 μm/s (**b**), and 750 μm/s (**c**,**d**) transient solute concentration along the *x*-direction at the positions ahead of the solidification front (along the red dashed lines in (**a**–**c**)).

Let us turn to the mechanism of the transition from the degenerate seaweed to tilted dendrite with the increase of the pulling velocity. Fig. 12(a–c) show typical quasi-steady-state interfacial morphologies for *V*_*p*_ = 200, 600, and 750 μm/s, respectively, at fixed thermal gradient *G* = 0.7 K/μm. In order to clearly reveal this morphological transition, the solid-liquid interfaces of these typical structures are extracted by plotting the contour of *ϕ* = 0. For the lowest case (*V*_*p*_ = 200 μm/s), the growth directions of the seaweed tips follow the thermal gradient direction with new branches forming at the tips. The weak surface tension anisotropy of the solid-liquid interface and the M-S instability on the tips are responsible for this processing. The lateral distribution of the solute concentration ahead of the solidification front of the degenerate seaweed is plotted in Fig. 12(d), and the red dashed line in Fig. 12(a) indicates the position of the cut line. It can be seen that the solute concentration curve irregularly oscillates in space, resulting from the non-periodic tip-splitting instability and irregular solidification front. For *V*_*p*_ = 750 μm/s, the morphology becomes dendritic arrays with a large tilted angle. As shown in Fig. 12(c), sidebranches develop far from the stable tip of the dendrite. Since the neighboring dendrite hinders their growth, sidebranches are too small to influence the growth of the dendritic tip. Although an asymmetrical dendrite gives rise to the asymmetrical lateral distribution of solute concentration (the red line in Fig. 12(d)), the variation of the solute concentration is regular and its maximum value is presented between the neighboring dendrites. For the intermediate case (*V*_*p*_ = 600 μm/s), we found that the interfacial morphology is more like a transitional state between the degenerate seaweed and the dendrite. It seems that the typical structure shown in Figure 12(b) has two “main” branches with their specific growth directions. The red arrows highlight the “main” branches in this degenerate seaweed. The tips of the “main” branches are more stable than that in the lowest case because the interfacial instability always occurs on the stalk of the “main” branch near the tip instead of the tips. The new branches from this instability are similar to the sidebranches in the dendritic arrays. However, because there exists sufficient space between the two main branches and the new branches are very close to their tips, the growth of the new branches can influence the growth of the “main tip” through ejecting the solute, which results in the irregular lateral distribution of the solute concentration ahead of the solidification front (the yellow line in Fig. 12(d)) and the alteration of the leading tip. This also gives rise to the oscillatory growth mode like the typical degenerate seaweed growth. Moreover, the tilted angle between the each main branch and the thermal gradient direction is about ±22.5°, which quantitatively agrees with the experimental and numerical finding in Reference^24^. Therefore, the place where the interfacial instability occurs determines the interfacial morphologies: the tip-splitting instability results in the typical degenerate seaweed, instability occurring close to the tip leads to transitional state degenerate seaweed, and instability occurring far behind the tip gives rise to the formation of the tilted dendrite.

**Figure 13:**
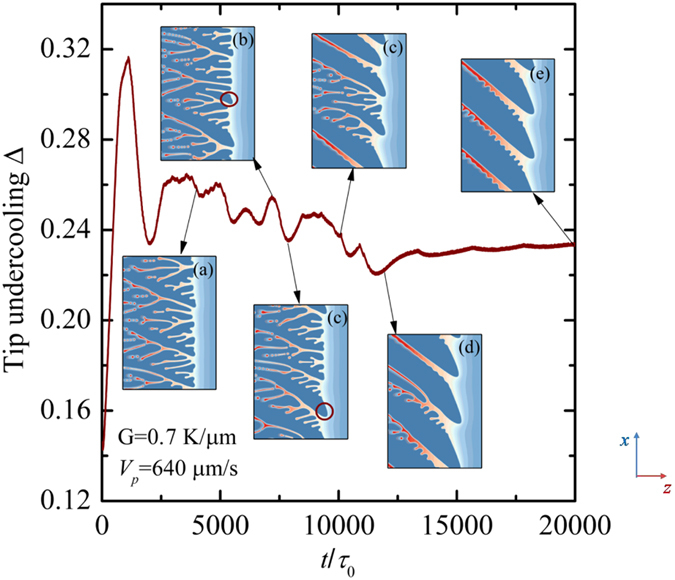
A representative example of the evolution of the dimensionless leading tip undercooling with time with the interfacial morphologies during the transient transition from degenerate seaweed to tilted dendrite for *V*_*p*_ = 650 μm/s and *G* = 0.7 K/μm.

In directional solidification, both degenerate seaweed and dendrite can be understood as two relatively stable growth patterns^25^. However, dendrites are dynamically preferred over degenerate seaweed because the degenerate is found to be unstable once the dendrite grows from it. Figure 13 shows a representative example of the evolution of the leading tip undercooling with time as well as the interfacial morphologies during the transition from degenerate seaweed to tilted dendrites for *V*_*p*_ = 650 μm/s and *G* = 0.7 K/μm. In the inserts, the red circles highlight the place where the dendrite first forms on the degenerate seaweed. It can be seen clearly that the degenerate seaweed become unstable once the tilted dendritic branch develops. There exists a competition between the degenerate seaweed and the tilted dendrite. Because the dendritic structures have lower undercooling, the degenerate seaweed cannot prevent the development of the dendrite. Furthermore, the formation of the new born dendrite can give rise to that an adjacent cell transits to a new dendrite because a higher constitutional undercooling region has been produced. This process is continued until the tilted dendrites are dominant (the insert (e) in Fig. 13). Because the periodic boundary conditions are used here, the tilted dendrites overgrow the degenerate very soon. For the evolution of the dimensionless leading tip undercooling in the transition from the degenerate seaweed to dendrite, one can see clearly that the dimensionless leading tip undercooling oscillates at higher values in the degenerate seaweed region while it has a lower value in the dendritic region.

In the morphological transition described above, under a certain value of thermal gradient, the degenerate seaweed structures become the tilted dendritic arrays with a large tilted angle once the pulling velocity exceeds a critical value. In the following part, we mainly focus on the characteristic of such tilted dendritic arrays in directional solidification: the primary spacing and the growth direction.

**Figure 14:**
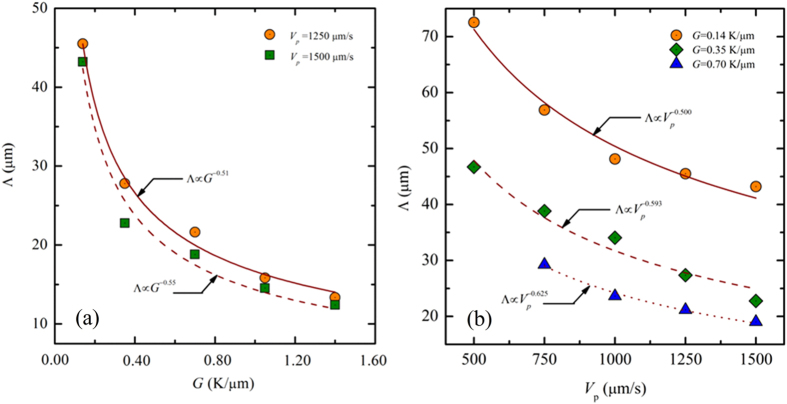
(**a**) Evolution of the primary spacing of the tilted dendritic arrays as a function of the thermal gradient *G* for *V*_p_ = 1250 and 1500 μm/s; (**b**) evolution of the primary spacing as a function of pulling velocity *V*_p_ for *G* = 0.14 K/μm, *G* = 0.35 K/μm and *G* = 0.7 K/μm.

Previous experimental and theoretical investigations^1^,^2^,^46^ show that the primary spacing varies with the thermal gradient as the power law Λ∝Δ*T*_0_^*a*^*V*_*p*_^−*b*^*G*^−*c*^ with *a* = *b* = 0.25 and *c* = 0.5 when the preferred growth direction of the dendritic arrays is aligned with the thermal gradient. Gandin *et al*.^31^ found that the primary spacing increases with the misorientation angle, following a classical power law as
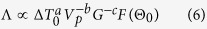
where 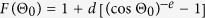 with *a* = *b* = 0.25, *c* = 0.5, *d* = 0.15 and *e* = 8 for SCN-3.61 wt.% acetone alloy. Very recently, Tourret and Karma^39^ confirmed this power law relationship by using phase-field simulations. However, limited studies focus on the tilted growth of dendritic arrays for Θ_0_ = 45°, the largest misoreintation angle in cubic system. A similar growth pattern with a large tilted angle has been seen in Reference^25^ although no mention is made of the primary spacing selection with respect to pulling velocity and thermal gradient. In our simulations, Θ_0_ is fixed, which means *F*(Θ_0_) should be fixed. Therefore, we mainly focus on the variation of the primary spacing with pulling velocity and thermal gradient varying. Figure 14(a) shows the evolution of the average primary spacing as a function of thermal gradient *G* for *V*_*p*_ = 1250 and 1500 μm/s. It can be seen that the behaviors of Λ versus *G* (Λ∝*G*^−*0.51*^ and Λ∝*G*^−*0.55*^ for *V*_*p*_ = 1250 and 1500 μm/s, respectively) from our predicted results are in a good agreement with Λ∝*G*^−*0.5*^ from experiments and numerical simulations^1^,^2^,^44^. Yet the primary spacing as a function of the pulling velocity follows the power law Λ∝*V*_*p*_^−0.50^, Λ∝*V*_*p*_^−0.593^and Λ∝*V*_*p*_^−0.50^ for *G* = 0.14 K/μm, *G* = 0.35 K/μm and *G* = 0.7 K/μm, respectively, as shown in Fig. 14(b). This is not consistent with Λ∝*V*_*p*_^−0.250^ form _*p*_revious work^1^,^2^,^31^,^39^,^44^ when Θ_0_<30°. The power law relationship Λ∝*V*_*p*_^−0.25^ given by the sim_*p*_le classical model implies that the local tip radius *R* has a behavior like *V*_*p*_*R*^2^ ~ constant while the _*p*_ower law Λ∝*V*_*p*_^−0.50^ in our simulations means that *V*_*p*_Λ^2^ is about a constant for this range of pulling velocity. We may attribute this difference to the different effective surface tension anisotropy in the direction of thermal gradient direction. Future work will focus on the role of the anisotropy strength on the primary spacing selection in directional solidification with nonzero misorientation angle, and the coefficients *d* and *e* of Equation (6) warrant further study in order to understand what alloy or control parameters affect them.

**Figure 15:**
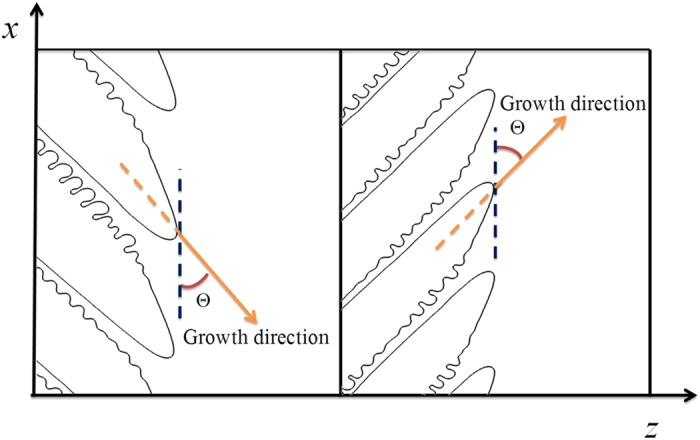
Schematic of the two definitions of tilted angle Θ in the growth of tilted dendritic arrays during directional solidification of non-axially oriented crystals.

**Figure 16:**
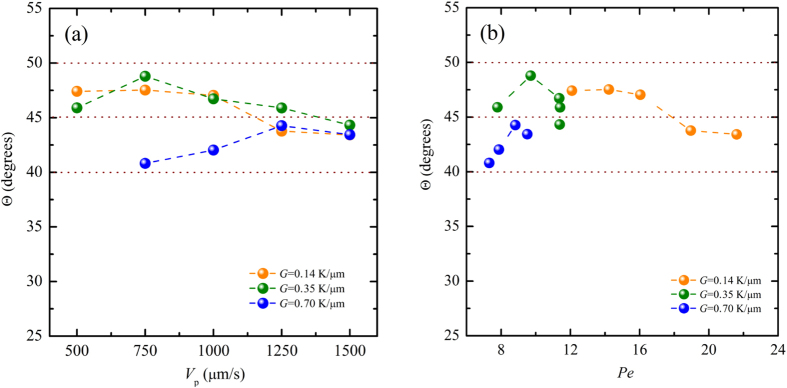
Variation of the tilted angle with the pulling velocity (**a**) and the Péclet number (**b**) for *G* = 0.14 K/μm, *G* = 0.35 K/μm and *G* = 0.7 K/μm.

Because a cubic system is considered here, the two preferred crystalline directions i.e. +45° (clockwise) and −45° (anti-clockwise) are illustrated in Fig. 1. In Fig. 6, the growth directions of the dynamically-stable dendritic arrays are either a clockwise tilt or an anti-clockwise tilt. According to present numerical data, we cannot justify the dependence of the clockwise and anti-clockwise on the thermal gradient and pulling velocity. The transient morphological evolution of the transition from degenerate seaweed to dendrite (Fig. 13) indicates that the initial tilted dendrite originates from the tips of degenerate seaweed. This is similar to the so-called “tail instability”. As shown in Fig. 12(b), the transitional state degenerate seaweed has two “main” branches that are symmetrical about the thermal gradient direction. Therefore, the clockwise and the anti-clockwise tilt of the dendritic arrays have the same possibility. Either the clockwise or the anti-clockwise tilt of the dendritic arrays is not explicitly assigned using the corresponding interfacial energy function. Indeed, there is no difference between the two types of the tilted dendritic arrays. For convenience to discuss the growth direction selection, the tilted angle Θ is defined as the angle between the growth direction and the *x*-axis positive or negative direction for the dendrites growing towards the two sides, respectively, as schematically shown in Fig. 15. Figure 16(a) shows the variation of the tilted angle with the pulling velocity for different values of the thermal gradient. It can be seen that all values of the tilted angles are found in the range from 40° to 50°. As the pulling velocity is increased, the growth directions for three different values of thermal gradient converge to a constant value (about 44°). Note that the anisotropy function is assumed as a four-fold anisotropy, Equation (4), in our simulations. It seems that if a dendrite grows at the tilted angle Θ > 45°, sidebranches growing at 90°−Θ < 45° will be favored. This may result in that sidebranches are dominant, and then the tilted angle is always less than 45°. However, we indeed observe that the tilted dendritic arrays grow at Θ > 45° in our simulations. Similar growth patterns have been observed in experiments of directional solidification of SCN-based transparent model alloys by Utter *et al*.^25^. In their opinions, a two-fold anisotropy could be important in such tilted dendrites with large growth angles but not the four-fold anisotropy. Our results indicate that under certain conditions, stable tilted dendrites can grow at Θ > 45° instead of the development of sidebranches at 90°−Θ *< *45° in other direction through two-dimensional phase-field simulations with a simple four-fold anisotropy. This is consistent with the observation shown in Fig. 25 of Reference^24^, where the [010] dendrite tilted at 53°. In general, the growth directions of cellular and dendritic arrays follow a continuous variation from the orientation of the thermal gradient to the preferred growth direction as the pulling velocity or the primary spacing increases for a lower misorientation angle. Different set of experiments have shown that the growth direction is a function of misorientation angle and Péclet number, *Pe* = *V*_*p*_Λ/*D*_*l*_, i.e., the DGP law^15^. Here, the variation of the dendritic growth angle with Péclet number for different values of thermal gradient is plotted, which is shown in Fig. 16(b). Clearly, the growth direction of dendritic arrays with large tilted angles is less sensitive to the pulling velocity and the primary spacing, and the DGP law cannot describe the growth direction selection of the tilted dendrites for Θ_0_ = 45°. Therefore, a new growth direction law is needed when Θ_0_ is relatively large in future work.

**Figure 17:**
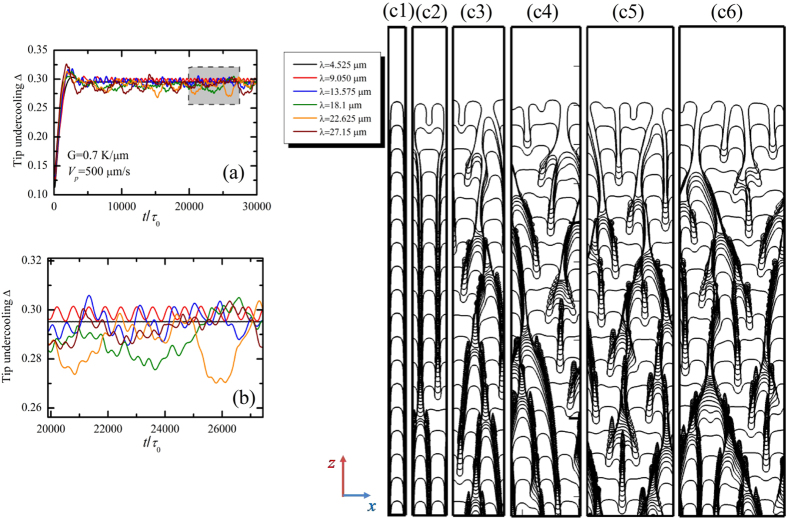
(**a**) Evolution of the dimensionless leading tip undercooling Δ for different values of initial interfacial wavelength for *V*_*p*_ = 500 μm/s and *G* = 0.7 K/μm; (**b**) is an enlarged view of (**a**,**c**) evolution of the interfacial morphologies, where (c1) to (c6) corresponds to Λ_*f*_ = 4.525 μm to 27.15 μm, respectively.

It is well known that a similar history results in a similar primary spacing in directional solidification of alloys^11^,^12^. Several experimental and numerical^13^,^14^,^31^ investigations have confirmed that a range of stable primary spacing can be selected for fixed thermal gradient and pulling velocity for Θ_0_ < 35°. For Θ_0_ = 45°, interfacial morphologies are more complex because there are two preferred growth directions for the growing solid. Hence it is necessary to investigate the role of the initial interfacial wavelength on the morphological patterns selections and their growth behaviors. Here, we provide insight into the history dependence of the morphological transition in the case of Θ_0_ = 45°. The evolutions of the interfacial morphologies with their dimensionless leading tip undercooling Δ for different values of initial interfacial wavelength ((c1) to (c6) corresponds to Λ_*f*_ = 4.525 μm to 27.15 μm, respectively) for *V*_*p*_ = 500 μm/s and *G* = 0.7 K/μm are shown in Fig. 17, where (b) is an enlarged view of (a). Although the solid originates from the initial perturbations added at the solid-liquid interface under the same solidification control conditions, the morphologies are strikingly different from the morphology at (0.7 K/μm, 500 μm/s) shown in Fig. 6. For the lowest wavelength (Λ_*f*_ = 4.525 μm), cells grow in the direction of the thermal gradient, and the leading tip undercooling is at a constant because the cellular tip is stable. In this case, cell elimination occurs since the initial primary spacing is rather small. This can be seen as an orientational ordered interfacial morphology. Doubled the initial wavelength (Λ_*f*_ = 9.05 μm), the stable cellular growth is broken, and a periodic tip-splitting instability occurs. As shown in Fig. 17(c2), the instability leads to an oscillatory cellular structure. It can be seen that with the growth of the cell, the tip first flattens, and then the tip-splitting instability occurs. The tip-splitting at its center gives rise to the two newly-born tips having the same growth speed. Yet the growth is still unstable, and the tip will become flatten with the appearance of the tip-splitting instability once again. The process is repeated, leading to the periodic tip-splitting and the formation of the oscillatory cellular arrays. In this case, the behavior of the leading tip undercooling shows simple sinusoidal shape with time (The red line in Fig. 17(a,b) ). This morphology can be seen as a transitional growth pattern between the orientational ordered state (cellular arrays) and the disordered state (degenerate seaweed). As the initial wavelength further increases, the periodic tip-splitting instability is broken and typical degenerate seaweed appears. Therefore, the interfacial morphology undergoes the orientational ordered state (cellular arrays, Fig. 17(c1)), transitional state (oscillatory cellular arrays, Fig. 17(c2)) and orientational disordered state (seaweed structures, Fig. 17(c3–6)) with the increase of initial interfacial wavelength.

**Figure 18:**
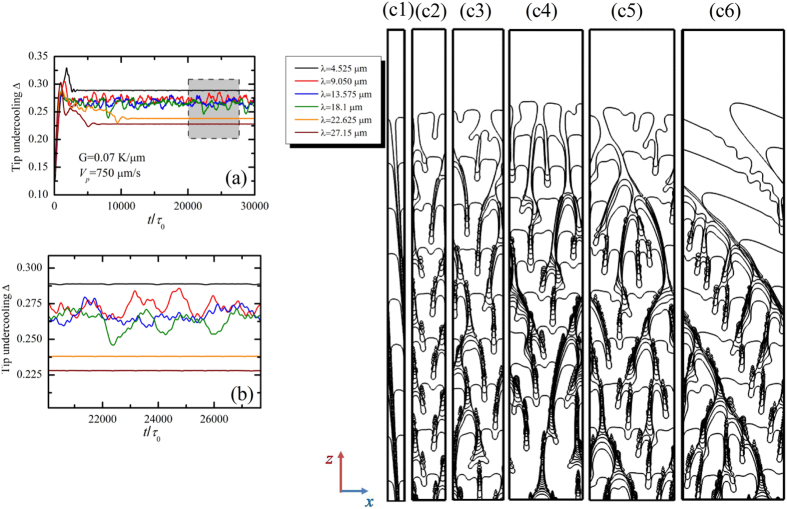
(**a**) Evolution of the dimensionless leading tip undercooling Δ for different values of initial interfacial wavelength for *V*_*p*_ = 750 μm/s and *G* = 0.7 K/μm; (**b**) is an enlarged view of (**a**,**c**) evolution of the interfacial morphologies, where (c1) to (c6) corresponds to Λ_*f*_ = 4.525 μm to 27.15 μm, respectively.

In additions, the pulling velocity is also an important factor in the history dependence of the morphological transition. In Fig. 6, the morphology becomes tilted dendritic arrays when randomly initial noises are introduced at the solid-liquid interface for *V*_*p*_ = 750 μm/s and *G* = 0.7 K/μm. Results are certainly different when the initial interfacial wavelength is set. Figure 18(a) shows the evolutions of the interfacial morphologies with their dimensionless leading tip undercooling Δ for different values of initial interfacial wavelength ((c1) to (c6) corresponds to Λ_*f*_ = 4.525 μm to 27.15 μm, respectively) when *V*_*p*_ = 750 μm/s and *G* = 0.7 K/μm, where (b) is an enlarged view of (a). For the lowest wavelength (Λ_*f*_ = 4.525 μm), the morphologies are cellular arrays whose growth direction deviates the direction of the thermal gradient with a constant leading tip undercooling. This is the main difference from that in the case of *V*_*p*_ = 500 μm/s, which indicates that the anisotropy begins to affect the growth direction as the pulling velocity is increased. As the initial wavelength increases, the morphological transition from tilted cellular arrays to degenerate seaweed to tilted dendtitic arrays occurs. In this transition, we also observe that the interfacial instability place is important for the morphological transition from degenerate seaweed to dendrite. As the initial wavelength increases, the interfacial morphology undergoes the tilted cellular arrays (stability, Fig. 18(c1)), the typical degenerate seaweed (instability occurs on the tip, shown in Fig. 18(c2, 3, 4)), the transitional state seaweed structures (instability occurs near the tip, shown in Fig. 18(c5)), and the tilted dendritic arrays (instability occurs far behind the tip, shown in Fig. 18(c6)).

## Conclusions

In this paper, we investigate the mechanism of the transition from degenerate seaweed to tilted dendrite and their growth dynamics during directional solidification of non-axially oriented crystals by using a thin-interface phase-field model in a two-dimensional system. A systematic convergence study for pulling velocity and anisotropic strength is carried out to choose an interface width small enough to ensure that the growth patterns in the numerical simulations are independent of the interface width. Morphological selection map in the plane of (*G*, *V*_*p*_) shows that the transition from degenerate seaweed to tilted dendrite occurs as the pulling velocity is increased or thermal gradient is decreased. In additions, the surface tension strength indeed plays an important role in the morphological transition, and the critical pulling velocity for the transition to dendrite follows a power-law relationship with respect to the anisotropy strength as *V*_p_^*^∝*ε*_4_^−1.4^. For the degenerate seaweed to the dendrite transition, either the increase of the interface thickness or the strength of the added arbitrary noise can result in the shift of the critical pulling velocity toward higher pulling velocity in the morphological maps in (*G*, *V*_*p*_) and (*ε*_4_, *V*_*p*_).

It is found that the dimensionless leading tip undercooling Δ can be used to characterize the oscillatory growth dynamics of the degenerate seaweed and distinguish the degenerate seaweed growth from the tilted dendritic growth. The dimensionless leading tip undercooling shows oscillations in degenerate seaweed growth while it keeps at a constant value in dendritic growth. This oscillation can be explained by the distribution of the liquid constitutional undercooling ahead of the solidification front. For the seaweed growth dynamics, the mechanism of the tip-splitting instability for Θ_0_ = 45° is different from that in the cellular growth for Θ_0_ = 0° since the anisotropy is the reason of the broader of the tip in the former case while it is responsible for the stability of the tip in the latter case. We found that the place where the interfacial instability occurs determines the morphological transition from degenerate seaweed to dendrite: the tip-splitting instability for the typical degenerate seaweed structures, the instability occurring close to the tip for transitional state degenerate seaweed, and the instability occurring far behind the tip for tilted dendritic arrays. In additions, the transient transition from the degenerate seaweed to the tilted dendrite reveals clearly that dendrites are dynamically preferred over seaweed because the dendrite has lower dimensionless tip undercooling under the same condition.

For the tilted dendrite with a large tilted angle in our simulations, it is found that the primary spacing follows a power-law relationship with respect to the pulling velocity as Λ∝*V*_*p*_^−0.50^ while following a power-law relationship with respect to the pulling velocity as Λ∝*G*^−*0.5*^. This indicates that the primary spacing of dendritic arrays with a larger tilted angle can be scaled by a similar power law. Our results indicate that under certain conditions, stable tilted dendrites can grow at Θ > 45° instead of the development of sidebranches at 90°−Θ < 45° in other direction through two-dimensional phase-field simulations with a simple four-fold anisotropy. This is consistent with the observation shown in Fig. 25 of Reference^24^, where the [010] dendrite tilted at 53°. Results show that the growth direction is less sensitive to the pulling velocity and the primary spacing, and hence the DGP law is invalid in the case of larger misorienation angles. Moreover, the effect of the initial interface wavelength on the morphological transition is also investigated to perform that morphological selections are history-dependent when Θ_0_ = 45°.

Noise plays an important role in the growth patterns selection and the increase of noise may lead to the transition from tilted dendrite to degenerate seaweed. Although we choose an interface width small enough to ensure the growth patterns independent of interface width, the residual grid noise cannot be quantitative. This is the limitation of the noiseless phase-field simulations. Future work should focus on the role of artificial noise on the formation of the degenerate seaweed structures and its growth dynamics because sufficient large noise could lead to the instability of the tip. The thermodynamic consistent thermal noise-induced concentration fluctuation in the liquid^46^ could be a choice to obtain more quantitative results. The role of the anisotropy strength on the primary spacing selection in directional solidification with nonzero misorientation angle should be discussed, and the coefficients *d* and *e* of Equation (5) warrant further study in order to understand what alloy or control parameters affect them. Furthermore, full three-dimensional phase-field simulations are necessary in order to investigate more complex growth shapes and the influence of the sample geometry on the morphological pattern selection by taking into account the interplay of different sources of anisotropies as discussed in Reference^41^.

Despite the limitations presented above, our numerical simulations provide insight into the mechanism of the transition from degenerate seaweed to tilted dendrite, showing the morphologies arising from varying the solidification conditions. Understanding the roles of solidification conditions and materials properties on the growth pattern selection is crucial for controlling the solidification microstructures and optimizing the mechanical properties of casting for a wide range of applications.

## Additional Information

**How to cite this article**: Xing, H. *et al*. Degenerate seaweed to tilted dendrite transition and their growth dynamics in directional solidification of non-axially oriented crystals: a phase-field study. *Sci. Rep.* **6**, 26625; doi: 10.1038/srep26625 (2016).

## Figures and Tables

**Table 1:** Parameters in the phase-field simulations of the morphological transition in the space of (*G*, *V*_*p*_).

	**λ**	***W***_**0**_
*V*_*p*_ = 100 ~ 200 μm/s	30	0.1697 μm
*V*_*p*_ = 500 ~ 1000 μm/s	20	0.1132 μm
*V*_*p*_ = 1250 ~ 1500 μm/s	10	0.0566 μm

**Table 2:** Parameters in the phase-field simulations of the morphological transition in the space of (*ε*_4_, *V*_*p*_).

	**λ**	***W***_**0**_
*ε*_4_ = 0.02 ~ 0.04	20	0.1132 μm
*ε*_4_ = 0.0075 ~ 0.01	10	0.0566 μm
